# *Cryptococcus neoformans* Is Resistant to Surfactant Protein A Mediated Host Defense Mechanisms

**DOI:** 10.1371/journal.pone.0001370

**Published:** 2007-12-26

**Authors:** Steven S. Giles, Aimee K. Zaas, Mike F. Reidy, John R. Perfect, Jo Rae Wright

**Affiliations:** 1 Department of Cell Biology, Duke University Medical Center, Durham, North Carolina, United States of America; 2 Department of Medicine, Duke University Medical Center, Durham, North Carolina, United States of America; University of Toronto, Canada

## Abstract

Initiation of a protective immune response to infection by the pathogenic fungus *Cryptococcus neoformans* is mediated in part by host factors that promote interactions between immune cells and *C. neoformans* yeast. Surfactant protein A (SP-A) contributes positively to pulmonary host defenses against a variety of bacteria, viruses, and fungi in part by promoting the recognition and phagocytosis of these pathogens by alveolar macrophages. In the present study we investigated the role of SP-A as a mediator of host defense against the pulmonary pathogen, *C. neoformans*. Previous studies have shown that SP-A binds to acapsular and minimally encapsulated strains of *C. neoformans*. Using *in vitro* binding assays we confirmed that SP-A does not directly bind to a fully encapsulated strain of *C. neoformans* (H99). However, we observed that when *C. neoformans* was incubated in bronchoalveolar fluid, SP-A binding was detected, suggesting that another alveolar host factor may enable SP-A binding. Indeed, we discovered that SP-A binds encapsulated *C. neoformans* via a previously unknown IgG dependent mechanism. The consequence of this interaction was the inhibition of IgG-mediated phagocytosis of *C. neoformans* by alveolar macrophages. Therefore, to assess the contribution of SP-A to the pulmonary host defenses we compared *in vivo* infections using SP-A null mice (*SP-A-/-*) and wild-type mice in an intranasal infection model. We found that the immune response assessed by cellular counts, TNFα cytokine production, and fungal burden in lungs and bronchoalveolar lavage fluids during early stages of infection were equivalent. Furthermore, the survival outcome of *C. neoformans* infection was equivalent in *SP-A-/-* and wild-type mice. Our results suggest that unlike a variety of bacteria, viruses, and other fungi, progression of disease with an inhalational challenge of *C. neoformans* does not appear to be negatively or positively affected by SP-A mediated mechanisms of pulmonary host defense.

## Introduction

*Cryptococcus neoformans* is an environmentally ubiquitous fungal pathogen that is responsible for significant morbidity and mortality in immunocompromised hosts. In humans, a pulmonary infection occurs following inhalation of *C. neoformans* cells. The identity of the infectious propagule remains unknown but is presumed to be either small, poorly encapsulated, desiccated yeasts or basidiospores. Hematogenous dissemination of *C. neoformans* from the lungs to the central nervous system can result in cryptococcal meningoencephalitis, a life-threatening complication that requires aggressive chemotherapeutic intervention [1]. In healthy hosts, initiation of an innate and adaptive cellular immune response limits the severity of the infection to an asymptomatic and often self-resolving pulmonary infection in most cases. The identification of those host factors that contribute to effective defenses against *C. neoformans* will not only broaden our understanding of *C. neoformans* pathogenesis but may aide in the development of therapeutic strategies for the prevention and treatment of this important fungal disease.

Surfactant protein A (SP-A) is one such innate host factor that has been shown to contribute positively to the pulmonary defenses against a diverse group of pathogenic microorganisms including, *Pseudomonas aeruginosa* [2], *Streptococcus pneumoniae* [2], *Respiratory Syncytial Virus* [3], *Influenza A* virus [4], and the fungi *Aspergillus fumigatus* [5] and *Pneumocystis jiroveci* [6]. SP-A mediates host defense via direct interactions between the carbohydrate recognition domain of SP-A and microbial ligands, which results in the opsonization and phagocytosis of pathogenic microorganisms by alveolar macrophages, and indirectly via modulation of the host immune response [7]–[9]. Previous *in vitro* studies have shown that SP-A can bind to acapsular and minimally encapsulated strains of *C. neoformans* but not heavily-encapsulated yeast [10], [11]. The role of SP-A in host defenses against *C. neoformans* remains to be fully elucidated.

In the present study we investigated the effect of SP-A on host defense against an encapsulated, pathogenic strain of *C. neoformans* (H99) [1]. The rationale for studying an encapsulated fully virulent strain was two-fold. First, acapsular strains of *C. neoformans* are uniformly avirulent, not clinically relevant and thus would not be suitable for investigating the role of SP-A as a mediator of host defense. Second, if a desiccated yeast form or basidiospore of *C. neoformans* were inhaled, rapid rehydration of capsule occurs in airway structures and the capsular structure is expected to be a major factor in the initial host-pathogen pulmonary interaction. We, therefore, initiated our study by first investigating the direct binding of SP-A to *C. neoformans* (H99). Using *in vitro* binding assays we confirmed that SP-A does not directly bind to *C. neoformans* but, we did discover an IgG dependent mechanism that does enable SP-A binding to *C. neoformans*. *SP-A-/-* mice and a murine cryptococcosis inhalation infection model were then used to investigate the role of SP-A in host defense against *C. neoformans*. These experiments revealed that SP-A does not appear to contribute to the pulmonary host defenses against *C. neoformans*.

## Materials and Methods

### Strains and media

*Cryptococcus neoformans* var. *grubii* strains H99 (serotype A, mating type alpha) was revived from 15% glycerol stocks stored at −80°C. H99 was maintained on yeast extract peptone dextrose (YPD; 1% yeast extract, 2% peptone and 2% dextrose) agar plates at 30°C. Prior to use in these studies, yeast were grown in YPD liquid medium overnight at 30°C, harvested, washed three times with sterile phosphate-buffered saline (PBS), suspended in phagocytosis buffer and counted with a hemacytometer to determine cell concentrations. For phagocytosis assays, *C. neoformans* were labeled with Alexa Fluor 647 (Invitrogen, Carlsbad, CA) per the manufacturers instructions. Stocks of Alexa Fluor 647 labeled yeast were maintained at −20°C until needed.

### Purification and Alexa Fluor 488 labeling of surfactant protein A

SP-A was isolated from the lavage fluid of alveolar proteinosis patients as previously described [12]. Briefly, the surfactant pellet from the lavage fluid was extracted with butanol. Butanol insoluble proteins were then resuspended in octylglucopyranoside (OGP) in 0.15 M NaCl. SP-A, which is insoluble in OGP and salt, was then suspended in Tris-buffered water, pH 7.4. Remaining OGP was removed by dialysis against 5 mM Tris-buffered water, pH 7.4. SP-A was then treated with polymyxin agarose beads to remove endotoxin as previously described [12]. All SP-A preparations were assayed for endotoxin contamination using the *Limulus amebocyte* lysate assay (BioWhittaker, Rockland, ME) per the manufacturer's instructions. SP-A was labeled with Alexa Fluor 488 (Invitrogen, Carlsbad, CA) per the manufactures instructions. The ratio of moles dye per mole of protein was quantified and SP-A preparations with labeling efficiencies between 4 and 10 were used for binding assays.

### Surfactant protein A binding assays

To assess SP-A binding to *C. neoformans*, yeast were treated with Alexa Fluor 488 labeled SP-A (5 to 20 µg/ml), anti-GXM IgG mAb 18B7 (1.0 µg/ml, generous gift of Arturo Casadevall) or a combination of SP-A and anti-GXM IgG for 60 minutes at 37°C with shaking. Following treatment, yeast were washed twice with 5 volumes of phagocytosis buffer (PB) (PBS +0.1% BSA +900 µM CaCl_2_) and binding was quantified by flow cytometric analysis (Duke University Human Vaccine Institute Flow Cytometry Core Facility, Durham, NC). Data analysis was performed using the FlowJo software package (Ver. 6.3.3, Tree Star, Inc., Ashland, OR). For fluorescence microscopy experiments, PE-Texas Red (Invitrogen, Carlsbad, CA) labeled anti-GXM IgG mAb 18B7 was used to detect *C. neoformans* capsule. Calcofluor was used to stain the cell wall. SP-A binding assays using *Escherichia coli HB101* were used to validate the binding activity of all SP-A preparations used in this study. Binding was visualized by fluorescent microscopy (Zeiss Axioskop 2 Plus microscope). Fluorescent images were captured using an AxioCam MRm (Carl Zeiss, Thornwood, NY) digital camera and AxioVision 4.5 software (Carl Zeiss, Thornwood, NY). Images were uniformly processed to adjust contrast and image clarity using Adobe Photoshop CS2.

### Uptake and binding assays

Alveolar macrophages were isolated from Sprague-Dawley rats (Taconic, Germantown, NY). Bronchoalveolar lavage (BAL) was performed with 10 ml of PBS containing 0.2 mM EGTA (instilled 10 times). Cells were washed once with PBS and resuspended in PB. Prior to the addition of Alexa Fluor 647 labeled *C. neoformans* to alveolar macrophages, yeast were incubated with SP-A (5 to 20 µg/ml), anti-GXM IgG (1.0 µg/ml) or a combination of SP-A and anti-GXM IgG at 37°C for 60 minutes with shaking. Yeast were washed twice with 5 volumes of PB and added to alveolar macrophages in suspension at a multiplicity of infection of 1∶1. Yeast and alveolar macrophages were co-incubated for 60 minutes at 37°C with gentle shaking and attachment/phagocytosis was quantified by flow cytometric analysis.

### In vivo testing

*C. neoformans* wild-type strain H99 was used to assess the immune response and susceptibility of *SP-A-/-* mice to infection by *C. neoformans*. The immune response to infection by *C. neoformans* was assessed by infecting groups consisting of 5 or 10 *SP-A-/-* or wild-type C57BL/6 mice via intranasal inhalation with 1×10^4^ or 1×10^5^ CFU of each strain (in a volume of 50 µl), as described previously [13]. The rationale for using 1×10^4^ yeast for studies that assessed the host immune response was so that we could measure the immune response to a relatively small number of *C. neoformans*. *SP-A-/-* and wild-type mice were infected with an inoculum of 1×10^5^ yeast, which is a common inoculum size used for susceptibility studies. Bronchoalveolar lavages (1 ml sterile PBS instilled times 3; recovery 0.8–0.9 ml) were performed and total lung tissues were collected for colony forming unit determination at days three and seven post-infection. These time points were chosen so as to capture early events in the immune response to *C. neoformans*. Mouse TNFα ELISAs (R&D Systems) were performed on bronchoalveolar lavage fluid per the manufacture's instruction. Cell differentials were performed by microscopic examination of cytospin preparations of BAL from mice seven days post infection. Slides were prepared with Wright-Giemsa stain per the manufacturers instructions. Susceptibility studies were performed with groups consisting of 5 *SP-A-/-* or wild-type C57BL/6 mice that were infected via intranasal inhalation with 1×10^5^ CFU of each strain (in a volume of 50 µl), as described previously [13]–[15]. Mice that appeared moribund (i.e. lethargic or exhibiting rapid weight loss) or in pain were euthanized. Mice were monitored twice daily and the mean time to mortality between the groups was determined. The Duke University Institutional Animal Use and Care Committee approved the animal protocol used for these experiments.

### Statistics

Statistical analysis was performed using the JMP statistics package (SAS, Cary, NC). Student's T-test or ANOVA was used to assess binding, phagocytosis and cell differential data for statistical significance. Survival statistics were performed to evaluate mouse survival data for statistical significance. *P*-values<0.05 were considered statistically significant.

## Results

### Antibody dependent SP-A binding to *C. neoformans*

**Figure 1:**
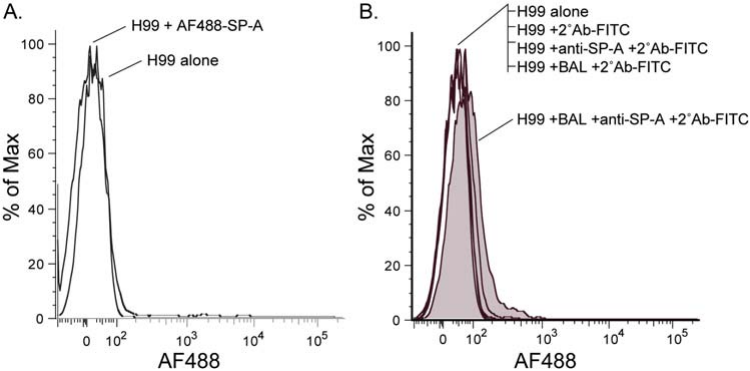
SP-A does not directly bind to encapsulated *C. neoformans* yeast. (A.) *C. neoformans* (1×10^5^) were treated with Alexa Flour 488 labeled SP-A (20 µg/ml) for 60 minutes at 37°C. Surfactant protein binding was quantified by flow cytometric analysis. SP-A binding was not detected. (B.) In contrast, *C. neoformans* (1×10^5^) treated with undiluted BAL exhibited a small amount of SP-A binding. Results are one representative experiment of at least three (A.) or two (B.) experiments performed.

Previous studies by Walenkamp et al. and Schelenz et al. showed that *in vitro*, SP-A binding is restricted to acapsular and minimally encapsulated strains of *C. neoformans* [10], [11]. To confirm these results, we performed flow cytometry to quantify AF488 labeled SP-A binding to encapsulated *C. neoformans* yeast (1×10^5^). SP-A binding assays using *Escherichia coli HB101* were performed to confirm the binding activity of all SP-A preparations (results not shown). Our results were consistent with those of previous studies; we did not observe SP-A binding to encapsulated H99 *C. neoformans* yeasts (Figure 1A). To extend this observation to biological fluids, *C. neoformans* yeasts (1×10^5^) were next treated with undiluted bronchoalveolar lavage fluid (BAL). In contrast to the previous conditions, we were able to detect a small amount of SP-A binding under these conditions (Figure 1B). Our interpretation of these results was that another factor present in BAL may have enabled SP-A to bind to *C. neoformans*. In the process of considering factors in BAL that might mediate SP-A binding to *C. neoformans*, immunoglobulins were selected as candidates for binding assays.

**Figure 2:**
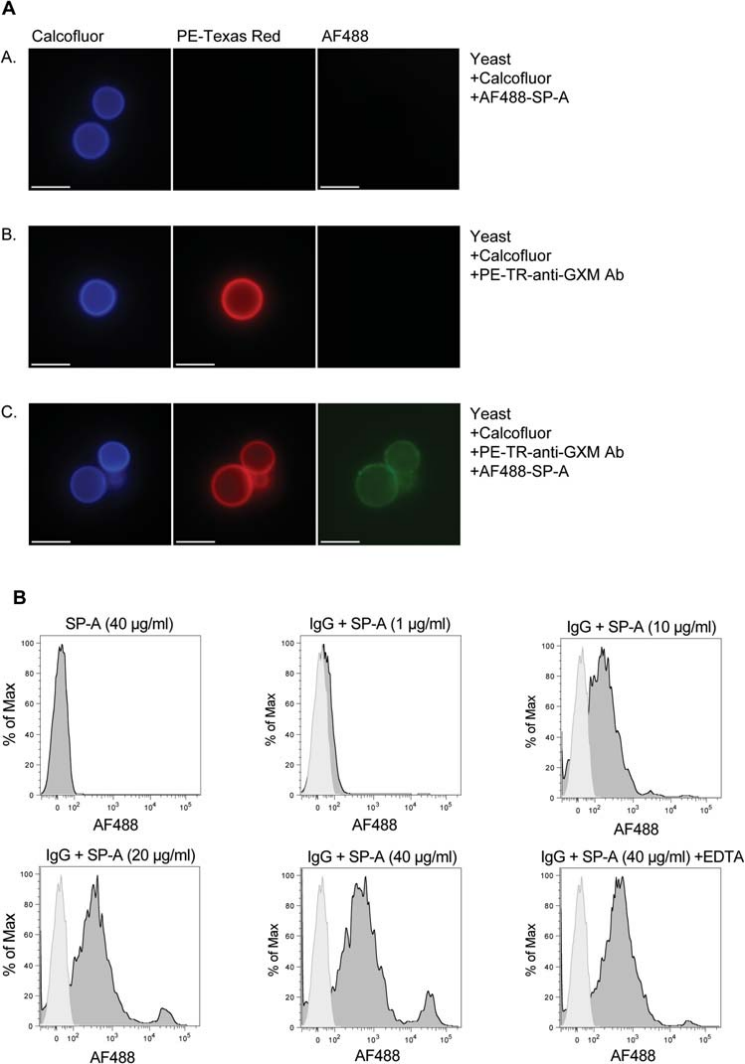
SP-A can bind *C. neoformans* via an IgG dependent mechanism. (A.) *C. neoformans* yeast (1×10^5^) were treated with AF488 labeled SP-A (Panel A), PE-Texas Red labeled anti-GXM IgG (Panel B) and PE-Texas Red labeled anti-GXM IgG and AF488 labeled SP-A (Panel C). Calcofluor was added to all samples to visualize the cell wall. SP-A binding to IgG opsonized (Panel C) but not non-opsonized (Panel A) *C. neoformans* was observed. Scale bar length is equal to 10 µm. (B.) IgG opsonized *C. neoformans* were treated with SP-A concentrations of 1, 10, 20, 40 µg/ml and binding was quantified by flow cytometric analysis. EDTA (1 mM) was included to determine if SP-A binding required the carbohydrate recognition domain. Histograms represent the mean fluorescence intensity of the AF 488 channel. At least 10,000 particles were interrogated for each sample.

Several studies have reported that naturally occurring IgG antibodies can non-specifically bind to pathogenic fungi. In addition, antibody binding can alter the physical properties of the *C. neoformans* capsule and affect the ability of other opsonins, such as complement to bind [16]. Therefore, we investigated whether IgG antibody binding to capsule enabled SP-A binding to *C. neoformans*. *C. neoformans* cells were opsonized with anti-GXM IgG and then treated with AF488 labeled SP-A (20 µg/ml) or left untreated as a control. Calcofluor was used to visualize the cell wall. While SP-A did not bind directly to *C. neoformans* cells (Figure 2A), the addition of anti-GXM IgG to the interaction resulted in robust SP-A binding that appeared to co-localize with anti-GXM IgG binding and was generally uniform throughout the capsular matrix (Figure 2A). Due to the size of the capsule, some areas bound by SP-A and anti-GXM IgG appear larger than the yeast. Further analysis by flow cytometry demonstrated that SP-A binding (1, 10, 20, and 40 µg/ml) to anti-GXM IgG opsonized *C. neoformans* cells occurred in a dose-dependent manner (Figure 2B). The carbohydrate recognition domain of SP-A is known to bind sugars in a calcium dependent manner. We observed that EDTA (1 mM) did not affect SP-A binding to anti-GXM IgG opsonized *C. neoformans*, suggesting that the carbohydrate recognition domain was not involved in this mechanism (Figure 2B). Furthermore, this result suggested that SP-A binding to anti-GXM IgG opsonized *C. neoformans* is not due to antibody mediated exposure of carbohydrate ligands.

### SP-A inhibits IgG dependent phagocytosis of *C. neoformans* by alveolar macrophages

**Figure 3:**
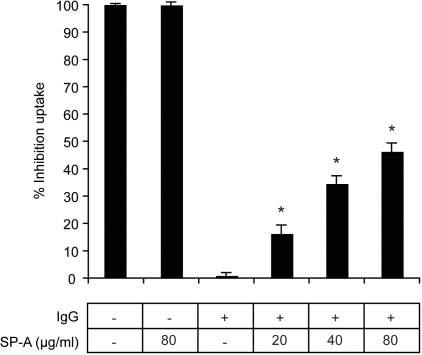
SP-A inhibits the uptake of IgG opsonized *C. neoformans* by alveolar macrophages. Non-IgG opsonized *C. neoformans* were attached/phagocytosed by less than 1% of the alveolar macrophage population in the presence or absence of SP-A, thus percent inhibition of uptake for these conditions is presented as ∼100%. SP-A (20, 40 and 80 µg/ml) significantly (*P*<0.001) inhibited the uptake of IgG opsonized *C. neoformans* (1×10^5^) by alveolar macrophages (1×10^5^) in a dose dependent manner (16±3.1%, 34±2.7% and 46±3.0%, respectively). Results represent the mean plus or minus the standard error of the mean of three experiments.

We next investigated whether SP-A binding could functionally affect IgG-mediated phagocytosis of *C. neoformans* by alveolar macrophages *in vitro*. For these experiments *C. neoformans* cells were fluorescently labeled with AF-647, which had no affect on *C. neoformans* uptake by alveolar macrophages in preliminary experiments (results not shown). Attachment/phagocytosis was quantified by flow cytometry. Non-IgG opsonized *C. neoformans* were attached/phagocytosed by less than 1% of the alveolar macrophage population in the presence or absence of SP-A, thus percent inhibition of uptake for these conditions is presented as ∼100% (Figure 3). We observed that all the SP-A concentrations tested (20, 40 and 80 µg/ml) significantly (*P*<0.001) inhibited the uptake of anti-GXM IgG opsonized *C. neoformans* yeast by alveolar macrophages and that this inhibition occurred in a dose dependent manner (16±3.1%, 34±2.7% and 46±3.0%, respectively) (Figure 3). These results demonstrate a unique mechanism by which SP-A inhibits, rather than enhances, the phagocytosis of an antibody opsonized microorganism by alveolar macrophages.

### *SP-A-/-* mice exhibit wild-type susceptibility to *C. neoformans*

Our *in vitro* studies suggested that the classic role of SP-A as an opsonin was unlikely to contribute to host defense against *C. neoformans*. In fact, its affect on IgG-mediated phagocytosis could enhance extracellular colonization and thus change the growth and dissemination of *C. neoformans in vivo*. Furthermore, SP-A is also known to contribute to host defense via other mechanisms, such as modulation of the immune response. Therefore, we could not predict the impact of SP-A on cryptococcosis. We explored the role of SP-A as a modulator of the early immune response against *C. neoformans* using a murine pulmonary infection model and an SP-A deficient mouse strain (*SP-A-/-*). Groups of *SP-A-/-* and wild-type mice were infected via intranasal inoculation with *C. neoformans* cells (1×10^4^) and several defined endpoints were analyzed at days three and seven post infection.

**Figure 4:**
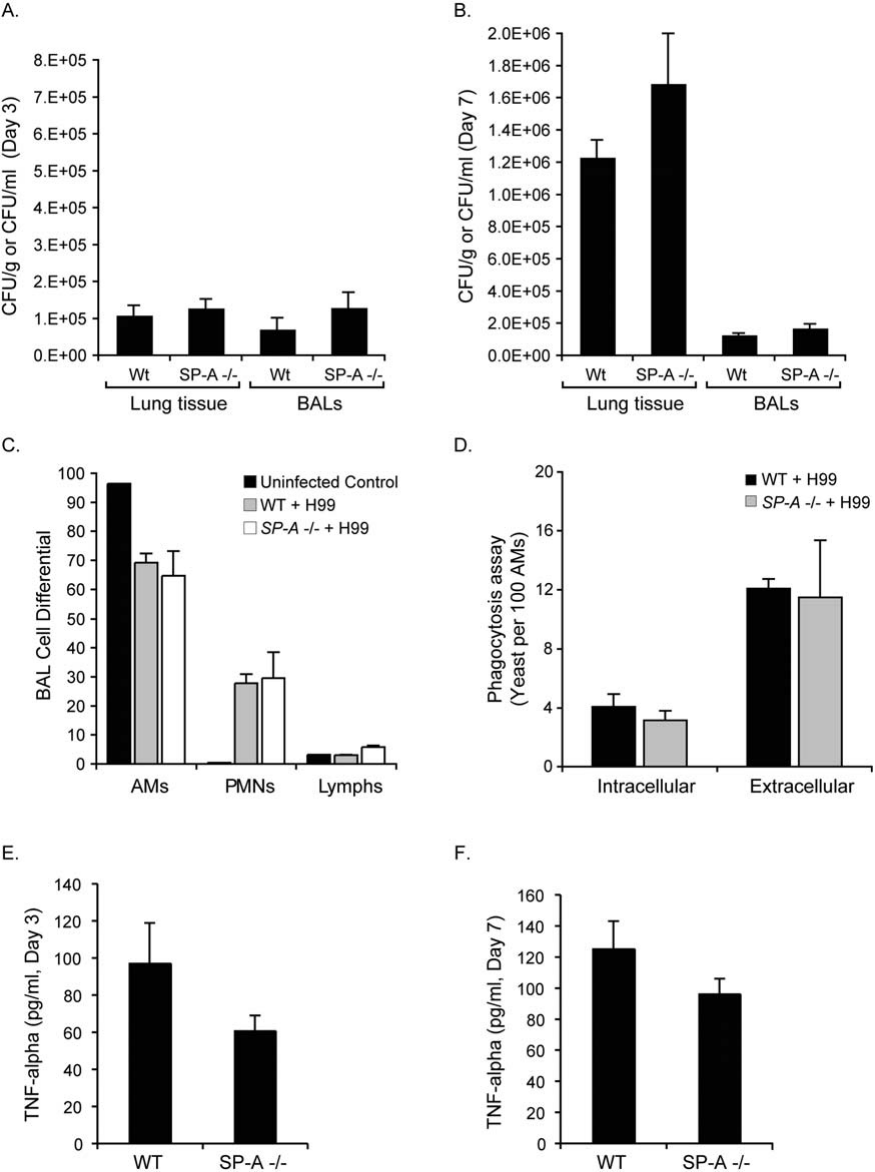
*C. neoformans* infection appears to progress equivalently in *SP-A-/-* and wild-type mice. (A.) Three days post infection the fungal burdens of lung tissue and BALs from wild-type (n = 4 and n = 7, respectively) and *SP-A-/-* (n = 3 and n = 8, respectively) mice were equivalent. (B.) A similar trend was observed at day seven post infection; no difference between groups were observed. (C.) Quantitative differentials showed no differences in the numbers of macrophages, polymorphonuclear neutrophils or lymphocytes present in BALs collected from wild-type (n = 3) and *SP-A-/-* (n = 3) mice seven days post infection. (D.) There were also no differences in the numbers of macrophages from *SP-A-/-* (n = 3) and wild-type mice (n = 3) that had phagocytosed *C. neoformans*. TNFα levels (measured by ELISA) in the lungs of wild-type and *SP-A-/-* mice at day three (n = 8 and n = 7, respectively) and seven (n = 16 and n = 17, respectively) post infection were equivalent (*P*>0.05). Results represent the mean plus or minus the standard error of the mean for the indicated number of experiments. (AM, alveolar macrophage; PMN, polymorphonuclear neutrophils; Lymphs, lymphocytes; BAL, bronchoalveolar fluid)

We began by first investigating whether SP-A deficiency affected the colonization of the lungs by *C. neoformans*. Bronchoalveolar lavage fluid and lung tissue were collected from *SP-A-/-* (n = 3 and n = 8, respectively) and wild-type (n = 4 and n = 7, respectively) mice at days three and seven post infection. We found that SP-A deficiency did not affect the colonization of the lungs by *C. neoformans* (Figure 4A). At day seven post infection a significantly greater (*P*<0.001) number of yeast were present in the lung tissue of both mouse strains, as compared to the bronchoalveolar lavage fluid (Figure 4B), suggesting that *C. neoformans* had equally invaded the pulmonary parenchyma of both strains.

We then investigated whether SP-A deficiency affected the recruitment of immune cells to the lungs in response to this infection in both murine strains. We found that the numbers of alveolar macrophages, polymorphonuclear cells or lymphocytes in the bronchoalveolar lavage fluid of *SP-A-/-* (n = 3) and wild-type (n = 3) mice at day seven day post infection were equivalent (Figure 4C), suggesting that SP-A deficiency did not affect the recruitment of immune cells in response to infection by *C. neoformans*. Furthermore, no significant (*P*>0.05) differences between the numbers of *C. neoformans* cells phagocytosed by alveolar macrophages from *SP-A-/-* (n = 3), as compared to wild-type (n = 3) mice was observed (Figure 4D). To investigate the impact on cytokine response to infection by *C. neoformans*, we measured a major Th1 cytokine, TNFα. We found that TNFα levels in bronchoalveolar lavage fluid from *SP-A-/-* and wild-type mice did not differ significantly at day three (n = 8 and n = 7, respectively) and seven (n = 16 and n = 17, respectively) post infection (Figure 4E and 4F). These results support the concept that SP-A deficiency did not affect TNFα production in response to infection by *C. neoformans*.

**Figure 5:**
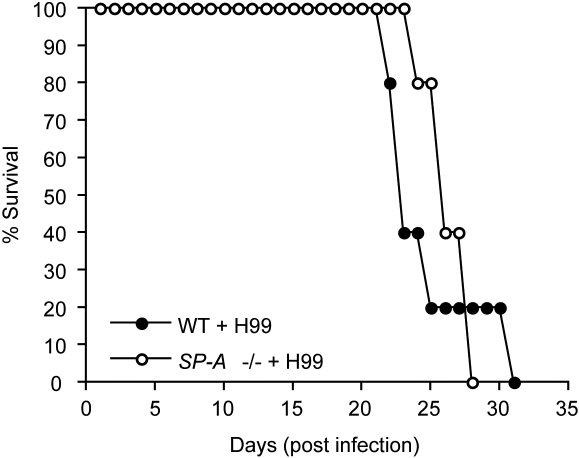
SP-A deficiency does not enhance susceptibility to infection by *C. neoformans.* Groups of 5 *SP-A-/-* and wild-type mice were infected with *C. neoformans* (H99, 1×10^5^) via intranasal inhalation. All mice succumbed to infection and died by day 32. There was no difference in the mean time to death of *SP-A-/-* and wild-type C57BL/6 mice.

As a final comparison of SP-A impact on infection, groups of 5 *SP-A-/-* and wild-type mice were infected by intranasal inoculation with *C. neoformans* yeast (1×10^5^) to investigate whether SP-A deficiency affected the final outcome of infection. By day 32 of infection all mice had succumbed to infection and died with no significant difference (*P*>0.05) in the mean time of mortality between the two groups (Figure 5). These results support the concept that SP-A deficiency does not affect the susceptibility of mice to infection by *C. neoformans*.

## Discussion

The inability of SP-A to bind encapsulated strains of *C. neoformans* is surprising given that SP-A binds a diverse assortment of carbohydrates and that the *C. neoformans* capsule and cell wall possess abundant and varied carbohydrates. Indeed, SP-A binds well to other pathogenic fungi including *Aspergillus fumigatus* [5], *Pneumocystis jiroveci* [17], *Coccidioides posadasii* [18], and *Histoplasma capsulatum* [19]. One explanation of these results may be found in studies that investigated SP-A binding to the yeast *Saccharomyces cerevisiae.* These studies found that despite the presence of accessible carbohydrates on the cell surface of *S. cerevisiae* SP-A does not bind to this yeast [20], [21]. This was hypothesized to be due to charge repulsion; SP-A and *S. cerevisiae* cell wall mannan both carry a net negative charge at pH 7.4 [21]. The capsular polysaccharide that surrounds *C. neoformans* yeast, which can reach diameters as large as 80 µm [22], [23] is very highly negatively charged [24]. It is possible that the absence of SP-A binding to *C. neoformans* is also due to charge repulsion. Since the concentrations of SP-A used in our *in vitro* binding assays were comparable or even higher than those thought to exist in the lungs of healthy individuals under physiological conditions (1 to 2 µg/ml) [11], it is unlikely that the binding deficiency was simply due to limited availability of SP-A.

This is the first report that has demonstrated IgG dependent binding of SP-A to a microorganism. Several lines of evidence suggest that this occurs via a mechanism in which IgG first binds to *C. neoformans* capsule and then SP-A binds to bound IgG. First, we showed that SP-A binding was not inhibited by EDTA, demonstrating that the SP-A carbohydrate recognition domain was not required. This ruled out the possibility of SP-A binding to carbohydrate ligands that may have been exposed as a result of anti-GXM IgG binding. Second, SP-A binding inhibited the IgG dependent phagocytosis of *C. neoformans* yeast by alveolar macrophages. This observation suggested that SP-A binding functionally interfered with IgG binging to the Fcγ receptor on the cell surface of alveolar macrophages, which is required for phagocytosis in the case of *C. neoformans* [1]. Third, a recent study in our laboratory showed that SP-A can bind directly to the Fc portion of IgG [25]. Since SP-A was used at physiologically relevant concentrations for these studies, our results suggest that this interaction could occur under *in vivo* conditions.

SP-A mediated mechanisms of host defense provide protection against several pathogenic fungi. For example, SP-A enhances the phagocytosis and killing of *Aspergillus fumigatus* conidia by alveolar macrophages and neutrophils [5]. In addition, administration of SP-A to mice with invasive aspergillosis rescued them from death [26]. Studies with *SP-A-/-* mice have shown that SP-A deficiency results in increased susceptibility to infection by *P. jiroveci* [27] and *H. capsulatum* [19]. However, this is not the case for *C. neoformans*. The end points analyzed in our *in vivo* studies clearly demonstrated an equivalent progression and outcome of disease in *SP-A-/-* and wild-type mice.

Another mechanism by which SP-A can mediate host defense is via direct modulation of alveolar macrophage function. SP-A can reduce reactive oxygen intermediate production by alveolar macrophages via the down regulation of NADPH oxidase [28]. Thus, alveolar macrophages in *SP-A-/-* mice would be expected to produce increased levels of reactive oxygen species as compared to wild-type mice. This up regulation of reactive oxygen species however would not be expected to have a significant effect on *C. neoformans* colonization of the lungs since *C. neoformans* possesses a highly robust antioxidant defense system [13]–[15] and the absence or presence of SP-A had no impact on yeast growth, host immune cell recruitment or cytokine production. Taken together, the *in vitro* and *in vivo* results of this study suggest that unlike a variety of bacteria, viruses and other fungi, the encapsulated yeast *C. neoformans* does not appear to be susceptible to SP-A mediated mechanisms of innate immunity in the murine pulmonary tract.
